# Locked nucleoside analogues expand the potential of DNAzymes to cleave structured RNA targets

**DOI:** 10.1186/1471-2199-7-19

**Published:** 2006-06-05

**Authors:** Birte Vester, Lykke H Hansen, Lars Bo Lundberg, B Ravindra Babu, Mads D Sørensen, Jesper Wengel, Stephen Douthwaite

**Affiliations:** 1 The Nucleic Acid Center, Department of Biochemistry and Molecular Biology, University of Southern Denmark, DK-5230 Odense M, Denmark; 2 The Nucleic Acid Center, Department of Chemistry, University of Southern Denmark, DK-5230 Odense M, Denmark; 3 Department of Chemistry, University of Copenhagen, Universitetsparken 5, DK-2100 Copenhagen, Denmark

## Abstract

**Background:**

DNAzymes cleave at predetermined sequences within RNA. A prerequisite for cleavage is that the DNAzyme can gain access to its target, and thus the DNAzyme must be capable of unfolding higher-order structures that are present in the RNA substrate. However, in many cases the RNA target sequence is hidden in a region that is too tightly structured to be accessed under physiological conditions by DNAzymes.

**Results:**

We investigated how incorporation of LNA (locked nucleic acid) monomers into DNAzymes improves their ability to gain access and cleave at highly-structured RNA targets. The binding arms of DNAzymes were varied in length and were substituted with up to three LNA and α-L-LNA monomers (forming LNAzymes). For one DNAzyme, the overall cleavage reaction proceeded fifty times faster after incorporation of two α-L-LNA monomers per binding arm (*k*_obs _increased from 0.014 min^-1 ^to 0.78 min^-1^).

**Conclusion:**

The data demonstrate how hydrolytic performance can be enhanced by design of LNAzymes, and indicate that there are optimal lengths for the binding arms and for the number of modified LNA monomers.

## Background

DNAzymes function as specific endonucleases by binding to predetermined sequences in RNA and cleaving its phosphodiester backbone. The discovery that RNA-hydrolytic properties could be encoded within a DNA oligonucleotide indicated potential biotechnological applications in gene silencing. These applications might even surpass those of other oligonucleotide-based gene silencing approaches, such as antisense and RNAi technologies, that require the complicity of the cell's own nuclease systems in order to cleave RNA. However, the use of DNAzymes has been restricted by several limitations, some of which are shared with the other oligonucleotide-based technologies. For any of these approaches to be of value, the oligonucleotide must be capable of transversing the cellular membrane and avoid being inactivated by cellular nucleases long enough to find the appropriate cellular compartment where it can bind and induce cleavage at the target RNA. Modification of DNAzymes to improve their stability against cellular nucleases and their ability to bind and cleave RNA molecules would go a long way towards increasing their general applicability.

**Figure 1:**
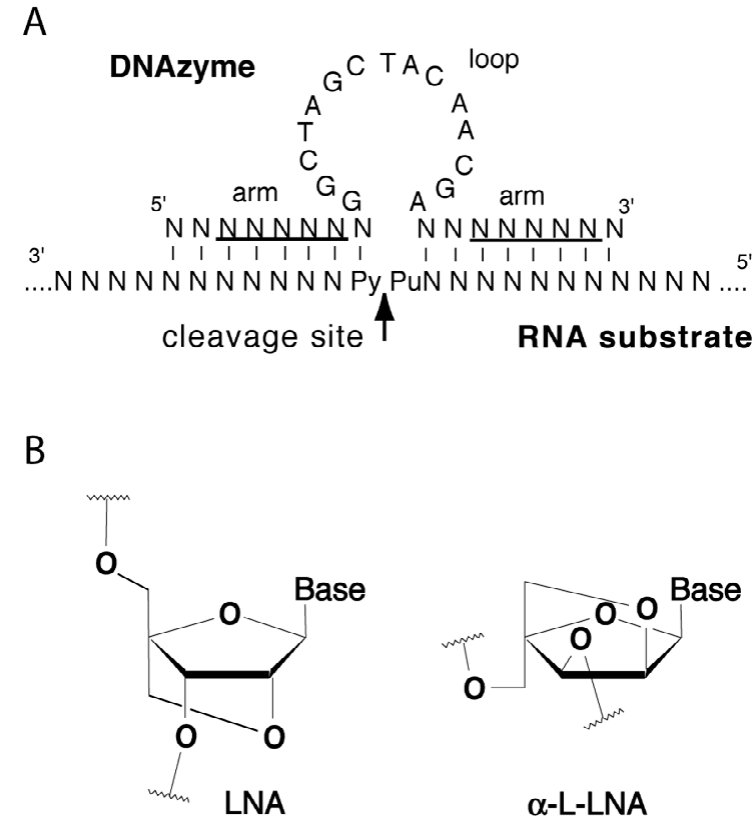
A. Schematic of a 10–23 DNAzyme hybridized via Watson-Crick base-pairing to an RNA substrate. The underlined sequence indicates the region where the LNA-type monomers were introduced (Table 1). Pu, purine; Py, pyrimidine. B. Structures of LNA (locked nucleic acid) and α-L-LNA nucleoside monomers.

The 10–23 DNAzyme isolated by Santoro and Joyce [1] (Fig. 1A) provides a suitable starting point for such modification and improvement. DNAzymes of this type cleave purine-uracil targets, and to a lesser extent purine-cytosine targets, and have been subjected to numerous investigations to alter target preference [2], to follow the folding of the catalytic region [3], and to induce cleavage at different targets [4,5]. Quite conclusive is that most DNAzymes of this type fail to cleave their RNA targets *in vitro *[4,5], and this does not bode well for *in vivo *application with the additional complications of cellular nucleases and substrate compartmentalization. Poor cleavage *in vitro *is generally linked to the inability of a DNAzyme to recognize its target, which involves unravelling the higher-order structure of the RNA and hybridizing via the two binding arms of the DNAzyme (Fig. 1). The binding arms can be constructed to include modified nucleosides, and such modifications have improved resistance against cellular nucleases [6-10]. In principle, the inclusion of modified nucleosides at specific sites in the binding arms could also greatly enhance the hybridization potential of DNAzymes at their RNA targets.

Modified nucleosides in the form of conformationally locked analogues (Locked Nucleic Acid, LNA) are capable of targeting complementary RNA and DNA with high affinity [11,12], and are thus potentially interesting as components in DNAzymes. Two commercially available variants of these modified nucleosides (LNA and α-L-LNA, Fig. 1B) can be incorporated into oligonucleotides to increase their hybridization capacity [13-15]. Recent reports [10,16-19] have shown that inclusion of LNA monomers into the binding arms of DNAzymes (forming what we term LNAzymes) enables structured RNA targets to be cleaved, even in cases where the targets are intractable to unmodified DNAzymes. These studies indicate that the potential of LNAzymes might be optimized by rational design to cleave at targets that are located in highly structured RNA substrates.

Here, we have conducted a systematic approach geared towards the rational design of LNAzymes based on the 10–23 DNAzyme structure. Targets were displayed in RNA substrates with progressively more stable structures to challenge the cleavage capacity of DNAzymes. The ability of the DNAzymes to unravel the RNA structures was investigated by varying the lengths and the modification content of the hybridization arms. RNA substrates that are essentially inaccessible to unmodified DNAzymes were cleaved with markedly higher efficiency when LNA or α-L-LNA monomers were incorporated into the binding arms. For each RNA target, there appear to be optima for lengths of the binding arms, as well as for the type and number of modifications they contain. While the improved ability of LNAzymes to gain access to structured targets is the main reason for their greater capacity to cleave RNAs, the modifications also appear to favourably affect other kinetic parameters.

## Results

### RNA substrates for cleavage

**Figure 2:**
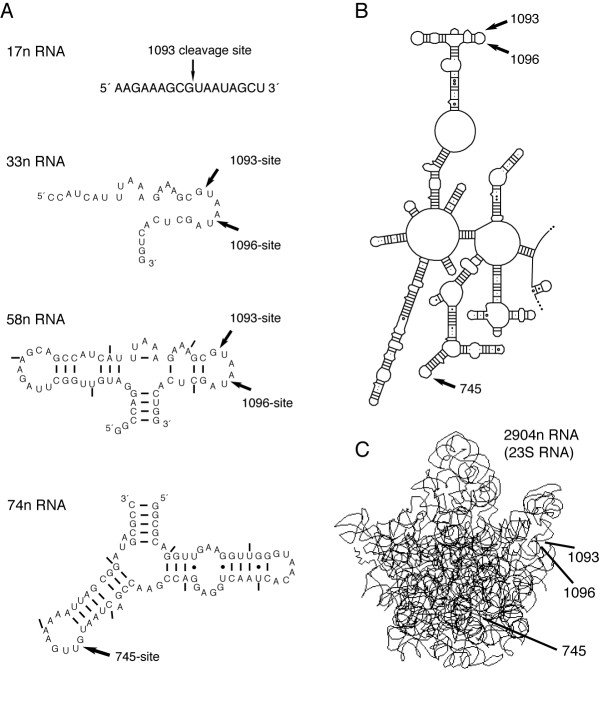
**RNA substrates for LNA- and DNAzyme cleavage**. RNA substrates for LNA- and DNAzyme cleavage. (A) 17n minimal RNA substrate; a 33n RNA with metastable secondary structure; and 58n and 74n RNAs that fold into well-defined secondary structures. Cleavage sites for the nucleic acid enzymes at nucleotides 745, 1093 and 1096 are shown by the arrows. The stem-loop secondary structures containing the cleavage targets have been verified by numerous techniques including biochemical probing [28], NMR [29] and crystallography [30-32]. (B) Secondary structure of domain II of 23S RNA (2904n RNA), which contains all three cleavage sites. (C) Higher-order folding of the rRNA (2904n) within the 50S ribosomal subunit; all the stem-loops in the smaller RNAs used here are maintained in the 2904n RNA (refs, PDB 1JJ2, [33]).

RNA substrates contain two consecutive sequences that are complementary to the binding arms of the DNAzymes and bracket the cleavage site between a purine and a pyrimidine (Fig. 1A). The cleavage sites studied here are displayed in RNA substrates that are based on sequences from the *Escherichia coli *23S ribosomal RNA, and possess a range of structural complexity (Fig. 2). The substrates consist of a 17n minimal RNA (largely unstructured), a 33n with metastable secondary structure, 58n and 74n RNAs with stable secondary structures, and a 2904n RNA (the intact *E. coli *23S rRNA) with extensive secondary and tertiary structure. Within these RNA substrates, three cleavage sites were studied: the 745-site (in the 74n and 2904n RNAs); the 1093-site (in the 17n, 33n, 58n and 2904n RNAs); and the 1096-site (in the 33n, 58n and 2904n RNAs).

### DNAzymes and LNAzymes

The nucleic acid enzymes used in this study range from unmodified DNAzymes to LNAzymes containing up to three LNA or α-L-LNA monomers per binding arm (Table 1). The lengths of the binding arms were also varied from five up to eight nucleotides; the catalytic loop remained unchanged in all the enzymes. A single DNAzyme and two LNAzymes were designed against the 745-site; one DNAzyme and one LNAzyme were made to cleave the 1096-site. The cleavage site at 1093 was studied most systematically, and two DNAzymes plus five LNAzymes were designed against this target. In the initial tests, the nucleic acid enzymes were used in molar excess relative to the RNA substrates, enabling us to study their performances under single-turnover conditions. The kinetic parameters of the most effective enzymes were then studied under multiple-turnover conditions with an excess of substrate.

### Cleavage at the 1093-site

**Figure 3:**
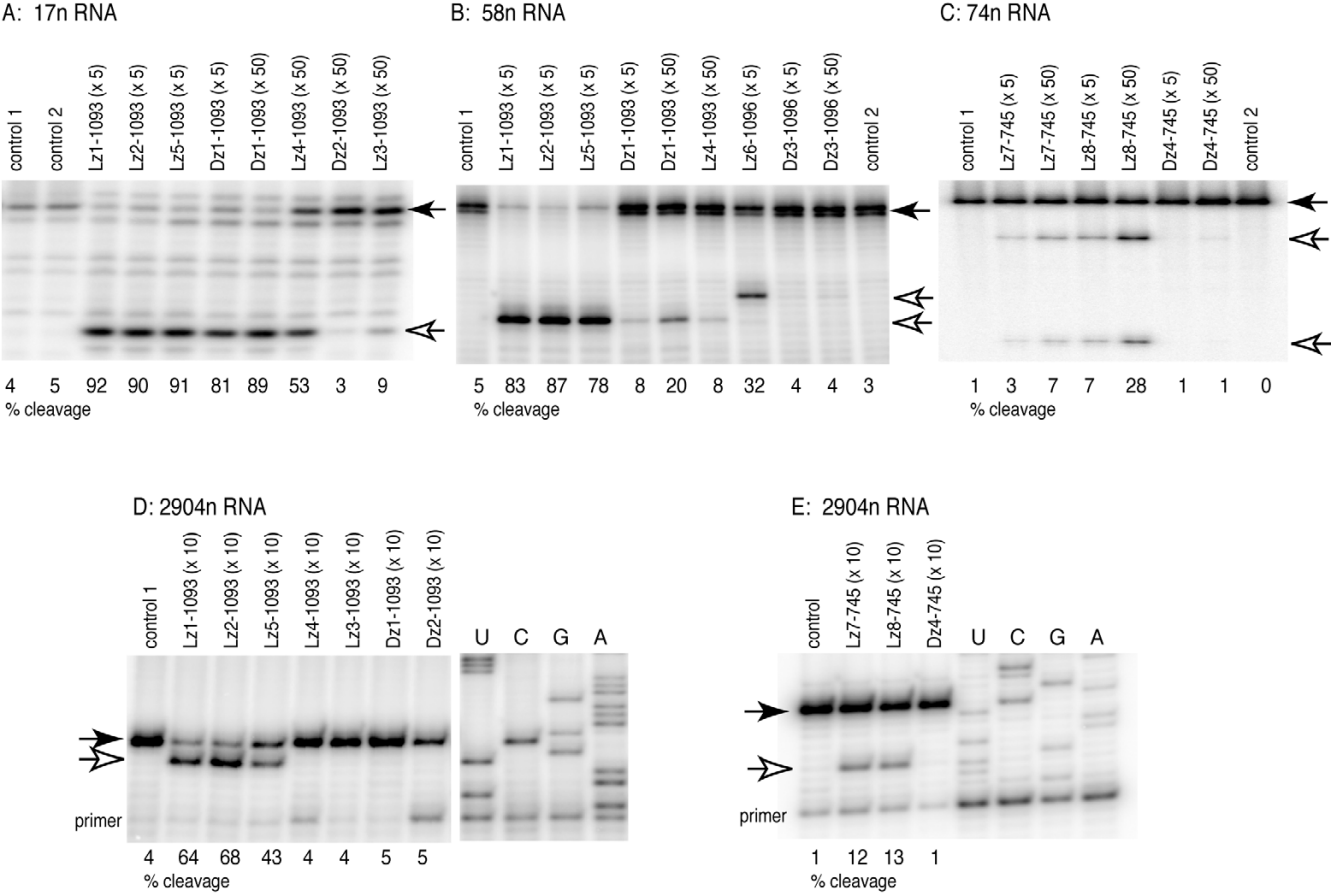
**Gel analysis of LNA- and DNAzyme cleavage of RNA substrates under single-turnover conditions**. The uncleaved RNAs substrates (filled arrows) and the cleavage products after one hour (unfilled arrows) are shown for a range of enzyme concentration (molar excess of enzyme in parentheses). No enzymes were added to the control sample, which were analyzed before (control 1) or after incubation (control 2). In A and B, the 17n and 58n RNAs are 5'-radiolabelled; in C, 74n is radiolabelled within the chain; in D and E, the radiolabel in on the 5'-end of the primer used in the reverse transcription reaction (and the bands observed here are thus cDNA transcripts of the 2904n RNA). The lanes U, C, G, and A denote dideoxysequencing reactions. The 58n RNA was partially extended with an extra 3'-nucleotide during T7 transcription (visible as a double band in the uncleaved substrate). The proportions of cleavage product are indicated below each gel lane.

Cleavage at the 1093-site in the 17n, 33n, 58n and 2904n RNAs was carried out under single-turnover conditions with a 5- to 50-fold excess of DNAzyme or LNAzyme (Fig. 3). The unstructured 17n RNA substrate was efficiently cleaved by the unmodified DNAzyme (Dz1-1093), and also by the corresponding LNAzymes with arms of eight nucleotides (Lz1-1093, Lz2-1093 and Lz5-1093) (Fig. 3A). However, when the 1093-site was displayed in the more tightly structured 58n RNA distinct differences were seen: cleavage by the unmodified DNAzyme was only marginal even at high concentration, whereas the LNAzymes still cut as effectively as in the 17n RNA (Fig. 3B). This picture was intensified in the more highly structured 2904n RNA, which the DNAzyme Dz1-1093 failed to cleave, whereas Lz1-1093 and Lz2-1093 still cut the 1093-site effectively (Fig. 3D). Incorporation of additional α-L-LNA modifications in Lz5-1093 resulted in less cleavage of 2904n RNA than with Lz1-1093 (Fig. 3D).

Upon reduction of the length of the binding arms to five and six nucleotides, the unmodified DNAzyme (Dz2-1093) lost all measurable hydrolytic activity and, even in large molar excess, was unable to cleave the minimal 17n substrate (Fig. 3A). The shorter arm size was counteracted to some extent by incorporating two α-L-LNA monomers into each arm (in Lz4-1093), resulting in good cleavage of 17n and moderate cleavage of 58n RNA (Fig. 3B). Further reduction of both arms to four nucleotides led to further loss of activity, and at 50-fold excess the LNAzyme Lz3-1093 could only weakly cut the 17n substrate despite having two α-L-LNA monomers per binding arm.

### Cleavage at the 745- and 1096-sites

These two additional cleavage sites were investigated to establish whether the observations made for the 1093-site could be extrapolated to other RNA targets. The 1096-site is particularly interesting in this context as it is located only three nucleotides away from the 1093-site and can thus be displayed in the same hairpin loop of the RNA substrates (Fig. 2). Despite this, the 1096-site proved more difficult to cleave. The DNAzyme Dz3-1096 failed to cut even when the 1096 target was presented in the relatively unstructured 33n RNA (and consequently also failed to cut the more complexly structured substrates, such as 58n RNA in Fig. 3B). Modifying the binding arms with two LNA monomers to form the LNAzyme Lz6-1096 led to effective cleavage at the 1096-site in the 33n and 58n RNA substrates (Fig. 3B).

The 745-site was also resilient to cleavage, and the unmodified DNAzyme Dz4-745 was incapable of cutting this target in either the 74n (Fig. 3C) or the 2904n RNAs (Fig. 3E). Some cleavage was achieved by modifying the enzyme to include two α-L-LNA monomers (Lz7-745) or two LNA monomers per binding arm (Lz8-745), but remained incomplete at 50-fold molar excess of the enzymes. The LNAzyme with LNA monomers was marginally, but consistently, better at cleaving the RNA substrates than its counterpart containing α-L-LNA.

### Kinetic parameters for cleavage at the 1093-site

**Figure 4:**
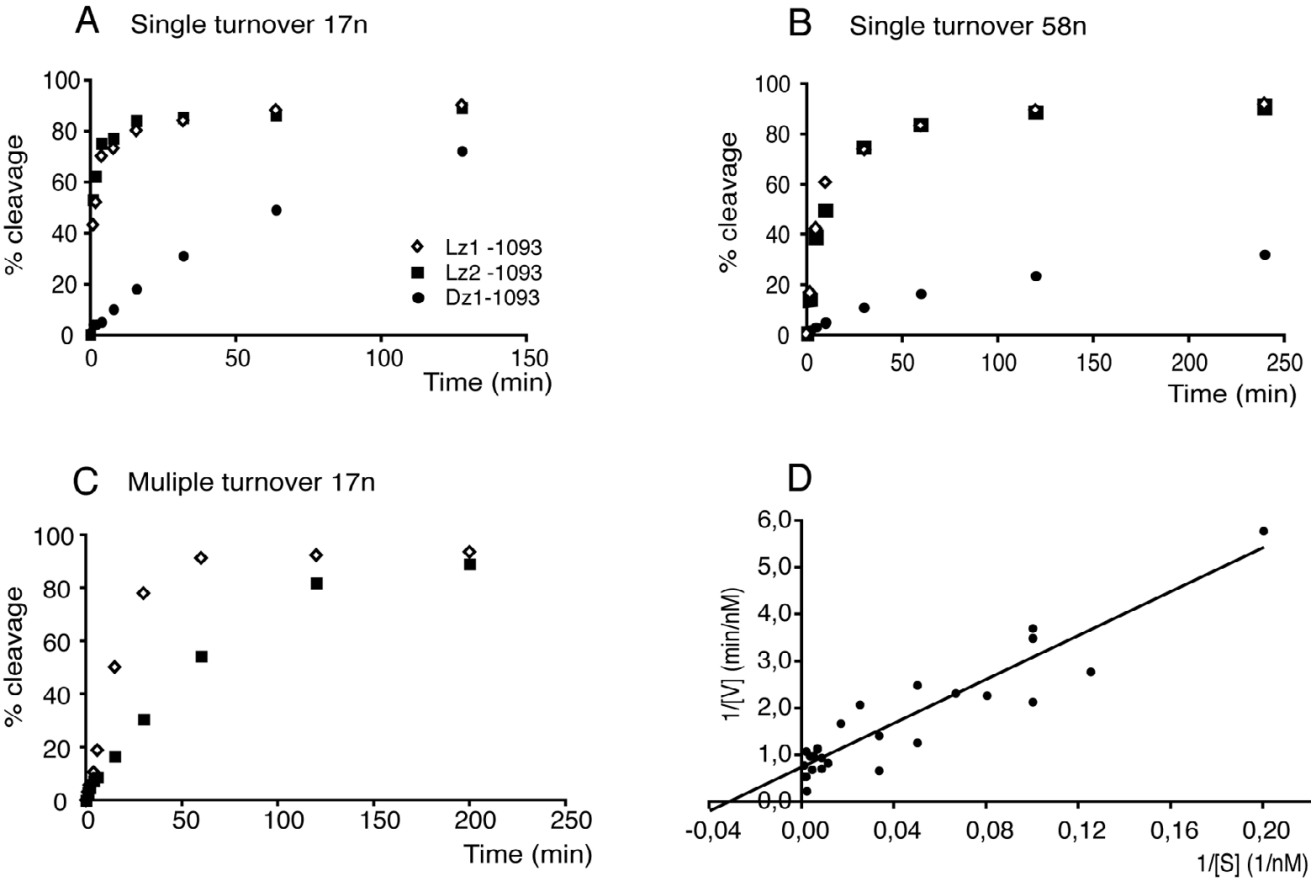
**Kinetics of cleavage at the 1093-site by LNAzymes**. (A) Single turnover cleavage of 17n by Dz1-1093 (circles), Lz1-1093 (squares) and Lz2-1093 (diamonds). The enzymes are in 5 × excess. (B) Single turnover cleavage of 58n by Dz1-1093 (in 50 × excess), Lz2-1093 (in 5 × excess), and Lz2-1093 (in 5 × excess). (C) Multiple turnover cleavage of 17n by Lz1-1093 and Lz2-1093. (D) Lineweaver-Burk plot showing Lz1-1093 data points obtained over a full range of substrate concentrations (5 nM to 1.2 μM), and used for determination of K_m _and V_max_.

The two LNAzymes Lz1-1093 and Lz2-1093 performed almost identically under single turnover conditions; this is illustrated in Figs. 4A and 4B and is consistent with a previous report [16]. The rate constant (*k*_obs_) for the unmodified Dz1-1093 and the LNAzyme Lz1-1093 was determined by fitting the time-course data for cleavage of the 17n RNA (Fig. 4A) into an exponential curve. This gave a *k*_obs _of 0.014 min^-1 ^for the Dz1-1093 and a *k*_obs _of 0.78 min^-1 ^for the Lz1-1093, showing that the overall reaction proceeded fifty times faster for the LNAzyme. It should be noted, however, that whereas the Dz1-1093 data fit comfortably into the exponential curve, the Lz1-1093 data fit much better into an equation describing a two step exponential decay. This suggests that the LNAzyme catalyzes a biphasic reaction, which has two *k*_obs _values of 1.37 min^-1 ^and 0.09 min^-1^.

The two most effective enzymes studied here, Lz1-1093 and Lz2-1093, cleave under a wide range of multiple-turnover conditions, and thus function in a truly enzymatic manner. Comparison of cleavage by the two LNAzymes in a 20-fold molar excess of the 17n substrate revealed that Lz1-1093 functions better than Lz2-1093 (Fig. 4C). Cleavage by Lz1-1093 under multiple turnover conditions was investigated further, and the results of time-course experiments for cleavage of the 17n RNA substrate at 5 nM to 1.2 μM are plotted in Fig. 4D. The initial reaction velocities (v) were measured from the time curves of cleavage at each substrate concentration; these were used in double reciprocal plots to estimate K_m _and the maximal velocity V_max_. Cleavage of the 17n RNA by Lz1-1093 proceeded with a K_m _of 33 nM and V_max _of 1.2 nM min^-1^; the Lz1-1093 LNAzyme concentration was 1 nM, giving a turnover number of 1.2 min^-1^. The unmodified Dz1-1093 did not cleave the substrate under these conditions.

## Discussion

Hybridization studies on oligonucleotides containing LNA and α-L-LNA monomers have shown that helical thermostability is greater than for unmodified nucleic acid chains [14]. Thus, we surmised that reconstructing DNAzymes to include LNA and α-L-LNA (forming LNAzymes) would improve their hybridization properties, and facilitate cleavage of obstinate RNA substrates. Consistent with this idea, incorporation of LNA and α-L-LNA monomers into the enzyme binding arms invariably improves cleavage performance, and this proved to be the case irrespective of whether the target was presented in an unstructured or complexly structured RNA substrate. This is consistent with other studies of LNA modified DNAzymes containing blocks of three to five LNA monomers at the ends of the binding arms [10,17]. Better hybridization of LNAzymes at the RNA target does indeed play a major role in enhancing cleavage, although other changes in kinetic parameters probably also contribute to the greater effectiveness of LNAzymes.

DNAzymes with eight nucleotides per binding arm were chosen as the starting point for our studies, as this arm length has been shown to provide a good balance between substrate recognition and product release [5,20,21]. Previous observations that only a small fraction of the potential purine-uridine sites can be cleaved by DNAzymes [4,5] were reflected in our choice of sites: one site (1093) is readily cleaved, whereas other two sites (745 and 1096) are intractable to DNAzymes.

Incorporation of LNA and α-L-LNA monomers into the binding arms of the DNAzymes improved cleavage at all three of the sites. Two LNA or α-L-LNA monomers per eight nucleotide binding arm worked best at the 1093-site, and increasing this number to three α-L-LNA monomers per binding arm (in Lz5-1093) caused a slight loss of activity (Fig. 3D), which was particularly noticeable on the 2904n RNA substrate. Heavily modified arms could adversely affect parameters such as nonspecific hybridization at other sites (especially in large substrates such as 2904n) and release of the product fragments after cleavage. However, product release effects would not be expected to come into play under single-turnover conditions with a large enzyme excess, so the extra modifications in Lz5-1093 must also interfere with other processes such as the accessibility of the LNAzyme's binding arms and/or the folding of its catalytic loop.

Reducing the lengths of the DNAzyme binding arms to five nucleotides led to loss of activity at the 1093-site (Dz2-1093). Activity was re-established by incorporating two α-L-LNA monomers into each of the arms (Lz4-1093). An LNAzyme with four nucleotides per binding arm, two of which were α-L-LNA monomers, also showed some cleavage activity (Lz3-1093). However, cleavage was only evident when the accessible 1093-site was displayed in the least structured substrate (17n) and challenged with a large molar excess of enzyme. Thus, the structure of the Lz3-1093 LNAzyme with its tetranucleotide arms is probably approaching the minimal size that can be expected to have hydrolytic activity.

LNA and α-L-LNA monomers in a DNA chain have markedly different effects on the helical geometry. Helices containing LNA monomers adopt a local A-form structure [13], whereas helices containing α-L-LNA tend more towards the B-form structure [15] with less pronounced stability [14]. In some cases it appeared more advantageous to use one type of modified monomer – for instance, at the 745-site, the LNA-enzyme was better than its α-L-LNA counterpart. At the more amenable 1093-site, however, no difference was seen in the single turnover cleavage by comparable LNA and α-L-LNA enzymes (Lz1-1093 versus Lz2-1093), nor did any differences appear when the 1093-site accessibility was reduced in the structured RNA substrates (58n and 2904n). The superior performance of Lz1-1093 compared to Lz2-1093 under multiple turnover conditions possibly reflects a difference in hybridisation energy that allows a faster product release from the α-L-LNA containing Lz1-1093 relative to Lz2-1093 with LNA monomers.

From the data presented here it can be seen that more highly structured RNA substrates have a general tendency to be more resilient to cleavage by nucleic acid enzymes, and that tighter structure can be counteracted by improving the hybridization capacity of the enzyme. However, several exceptions to this rule indicate that factors additional to substrate structure and enzyme hybridization play important roles in the cleavage process. For instance, the 1096-site was more difficult to cleave than the 1093-site despite being only three nucleotides away and being displayed in identical substrates. DNAzymes and LNAzymes designed against the 1096-site had binding arms that were expected to hybridize at least as strongly as the enzymes against the 1093-site (Dz3 and Lz6 are calculated to have Tm values when bound to the RNA that are 4°C higher than Dz1 and Lz2, respectively). Cleavage at the 1096-site occurs between A and U whereas cleavage at the 1093-site is between G and U. The identity of the purine can affect cleavage [22], although this does not fully explain the large difference observed in Figure 3B.

The kinetic measurements at the 1093-site indicate that several parameters are involved in determining the rate of the cleavage reaction. The apparent rate constant *k*_obs _for cleavage of the 17n substrate by the LNAzyme Lz1-1093 was fifty times greater than for the DNAzyme Dz1-1093 under single-turnover conditions with five times excess of enzyme. Although the excess of Dz1-1093 was probably not enough to saturate the target, such large differences in *k*_obs _would not be expected if this were the only factor limiting the reaction. Thus, the LNA residues in the binding arms probably influence parameters other than hybridisation. One such parameter could involve the folding of the catalytic loop into an active conformation. The biphasic reaction of Lz1-1093 in itself suggests the existence of two conformations of the LNAzyme and/or two conformations of the LNAzyme/substrate complex.

## Conclusion

In conclusion, we concur with Breaker's assertion that there is potential for improving the catalytic capacity of 10–23-like DNAzymes [23]. We show here that the ability of 10–23 DNAzymes to hybridize to their RNA substrates is an important factor in determining whether the target is cleaved, and in doing so we have improved the hybridization potential of several DNAzymes by the incorporation of locked nucleoside monomers into their binding arms. However, hybridization capacity is not the only parameter that can be altered in this way, and the apparent increased rate constant of the LNAzymes indicate that subsequent steps such as folding of the enzyme's catalytic loop have also, if somewhat fortuitously, been enhanced. The K_m _of 33 nM and the turnover number of 1.2 min^-1 ^measured for Lz1-1093 show that the binding affinity and *k*cat of this LNAzyme are approaching values that are suitable for practical applications.

## Methods

### DNAzymes and LNAzymes, 17n RNA, templates and primers

DNAzymes composed of unmodified deoxyoligonucleotide were obtained commercially. Modified DNAzymes with α-L-LNA- or LNA thymine monomers in the binding arms (LNAzymes) were synthesized using published procedures [11,14].

### RNA substrates

The 17n RNA was chemically synthesized (MWG Biotech AG). The 33n, 58n and 74n RNAs were transcribed using T7 RNA polymerase (Promega) using buffer conditions recommended by the supplier, and 1 mM nucleoside triphosphates. The following deoxyoligonucleotides were used as transcription templates: 33n, AATGATGGCTGCTTCTAAGCCAACATCCTGGCCTATAGTGAGTCGTATTA; 58n, CCAGTGAGCTATTACGCTTTCTTTAAATGATGGCTGCTTCTAAGCC AACATCCTGGCCTATAGTGAGTCGTATTA; 74n, GGCGCATCCGCTAATTTTTCAACATTAGTCCGTTCGGTCCTCCAGTAGTGTTACCCAACCTTCAACCTGCTCCTATAGTGAGTCGTATTA. All sequences were hybridized to the complementary deoxyoligonucleotide TAATACGACTCACTATAGG to form the double-stranded T7 promoter.

After transcription, the RNA were extracted and precipitated and the full-length RNA was isolated on a 13% denaturing polyacrylamide-7M urea gel. RNAs were eluted with H_2_O from gel bands, were extracted with phenol/chloroform, and were recovered by ethanol precipitation before being redissolved in H_2_O. RNA transcripts were generally 5'-radiolabelled by dephosphorylating using Shrimp phosphatase (USB) followed by incubation with T4 polynucleotide kinase (New England Biolab) and [γ-^32^P]-ATP. Alternatively, RNAs were labelled in the chain by inclusion of [α-^32^P]-CTP in the transcription reaction. The 2904n RNA (23S rRNA) was extracted from ribosomes isolated from *Escherichia coli *strain IB10 [24] using previously published methods [25]; the 2904n was not radiolabelled.

### RNA cleavage conditions for single and multiple-turnover analyses

The enzymes and RNA substrates were allowed to pre-equilibrate in the reaction buffer containing 150 mM NaCl and 50 mM Tris. HCl, pH 7.5 at 37°C for 5 minutes. The reaction was then initiated by addition of MgCl_2 _to a final concentration of 10 mM. In the single-turnover experiments in Figure 3, the concentration of the substrate RNA was 1 pmol in a reaction volume of 8 μl (125 nM). The deoxyribozyme was in excess at either 5 pmol or 50 pmol (625 nM or 6.25 μM) in experiments with the small RNAs, and at 10 or 20 pmol (1.25/2.5 μM) in experiments with the 2904n RNA. Single-turnover reactions with minimal RNAs were stopped after 1 h by quenching with one-third volume of ice-cold 90% formamide/20 mM EDTA. Samples were heated at 80°C for 2 min prior to loading onto 13% acrylamide polyacrylamide-7M gels.

The 23S rRNA was heated at 70°C for 30 sec and slow cooled to 37°C to enable refolding to its natural conformation before addition of the DNAzymes. Single-turnover reactions on the 2904n RNA were stopped by the addition of 20 mM EDTA at 0°C, and analyzed by primer extension as described below. RNA cleavage was detected by scanning the gels using phosphor imaging.

In the single-turnover experiments in Figure 4A and 4B, the concentration of the substrate RNA was 0.8 pmol in a reaction volume of 8 μl (100 nM) and the deoxyribozyme was at 4 pmol. In the multiple-turnover experiments shown in Figure 4C, the enzyme concentration was 30 nM while the substrate concentration was 0.6 μM. In the multiple-turnover experiments shown in Figure 4D, the enzyme concentration was kept constant at 1 nM while the substrate concentration was varied between 5 nM and 1.2 μM. Enzymes were pre-equilibrated with the ^32^P-labelled 17n RNA substrate in 150 mM NaCl, 50 mM Tris. HCl, pH 7.5 and 0.01% SDS at 50°C for 2 min followed by a further 2 min at 37°C, before the reaction was initiated by addition of MgCl_2 _to 10 mM. Sample aliquots were removed during the reaction and quenched prior to gel analysis (as described above).

### Analysis of cleavage in the 2904n RNA

Due to the large size of this RNA substrate, reverse transcriptase primer extension [26] was used to analyze cleavage. Briefly, AMV reverse transcriptase (Finnzymes) was used to extend two 5'-^32^P-end-labelled primers, 5'-CAAGTCATCCGCTAATTTT and 5'-GCCGACTCGACCAGTGAGC, complementary to 23S rRNA nucleotides 750 to 768 and 1099 to 1117, respectively, that are located immediately downstream from the cleavage sites. Transcripts stop either at the site of cleavage or immediately after on uncleaved RNA molecules due to incorporation of a dideoxynucleotide [27]. Extension products were analyzed on 13% polyacrylamide/7M urea gels alongside the sequencing reactions performed on uncleaved RNA [25].

## Authors' contributions

MDS and BRB performed the chemical synthesis of the LNAzymes. JW helped design and supervised the synthesis of the LNAzymes. LBL and LHH carried out the cleavage experiments. BV and SD conceived and designed the study, and wrote the manuscript. All authors read and approved the final manuscript.

## Figures and Tables

**Table 1:** DNAzyme and LNAzyme sequences and their RNA substrates. The catalytic sequence (loop) is invariable in all the nucleic acid enzyme constructions and has the sequence 5'-GGCTAGCTACAACGA. RNA modifications in the hybridization arms are indicated by bold underlined nucleotides; the cytidine LNA residue in Lz6-1096 is methylated at the 5-position of the base. The RNA substrates that were cleaved by the respective enzymes are underlined, those that could not be cleaved are italicized (the enzyme:substrate ratios for cleavage are defined in Fig. 3).

**Enzyme code**	**Sequence (5' to 3')**	**Type of modification**	**RNA substrates **(cleaved*uncleaved*)
Dz4-745	TTTTTCAA loop ATTAGTCG	none	*74n, 2904n*
Lz7-745	TT**T**T**T**CAA loop AT**T**AG**T**CG	α-L-LNA	74n, 2904n
Lz8-745	TT**T**T**T**CAA loop AT**T**AG**T**CG	LNA	74n, 2904n
Dz1-1093	AGCTATTA loop GCTTTCTT	none	17n, 33n, 58n,*2904n*
Lz1-1093	AGC**T**AT**T**A loop GC**T**T**T**CTT	α-L-LNA	17n, 58n, 2904n
Lz2-1093	AGC**T**AT**T**A loop GC**T**T**T**CTT	LNA	17n, 58n, 2904n
Lz5-1093	AGC**T**A**TT**A loop GC**T**T**T**C**T**T	α-L-LNA	58n, 2904n
Dz2-1093	TATTA loop GCTTTC	none	*17n*, *2904n*
Lz4-1093	**T**AT**T**A loop GC**T**T**T**C	α-L-LNA	17n, 58n, 2904n
Lz3-1093	A**TT**A loop GC**TT**	α-L-LNA	17n, *58n*, *2904n*
Dz3-1096	GTGAGCTA loop TACGCTTT	none	*33n*, *58n*
Lz6-1096	GTG**A**GC**T**A loop TA**C**GC**T**TT	LNA	33n, 58n
